# Point-of-sale sugar-sweetened beverage warning posters *v*. control posters were associated with reductions in school store sugar-sweetened beverage purchases made by Guatemalan adolescents

**DOI:** 10.1017/S1368980025101638

**Published:** 2026-01-02

**Authors:** Violeta Chacón, Joaquín Barnoya, Laura Gibson, Sophia Mus, José Carlos Monzón Fuentes, Alisa Stephens, Marsha Trego, Caitlin Lowery, Christina Economos, Alison Tovar, Sara C. Folta, Christina Roberto

**Affiliations:** 1 Rudd Center for Food Policy and Health, University of Connecticut, Hartford, CT 06103, USA; 2 Universidad Rafael Landívar, Guatemala City, Guatemala; 3 Ministerio de Salud Pública y Asistencia Social, Guatemala City, Guatemala; 4 Department of Medical Ethics and Health Policy, University of Pennsylvania Perelman School of Medicine, Philadelphia, PA, USA; 5 Department of Biostatistics, Epidemiology and Informatics, Center for Clinical Epidemiology and Biostatistics, University of Pennsylvania Perelman School of Medicine, Philadelphia, PA, USA; 6 Department of Nutrition, University of North Carolina at Chapel Hill, Chapel Hill, NC, USA; 7 ChildObesity180, Friedman School of Nutrition Science and Policy, Tufts University, 150 Harrison Ave, Boston, MA, 02111, USA; 8 Department of Behavioral and Social Sciences, Brown University, Providence, RI 02912, USA

**Keywords:** Sugar-sweetened beverages, Low- and middle-income countries, Food labelling, Front-of-package, Nutrition labelling

## Abstract

**Objective:**

We examined whether point-of-sale warning posters, compared with control posters, reduced Guatemalan adolescents’ purchases of sugar-sweetened beverages (SSB) at school stores.

**Design:**

We used a difference-in-differences approach (4-week baseline and 4-week treatment). Our primary analysis compared two schools assigned to an intervention warning poster to one school that displayed a control poster. Based on purchase transaction data, the outcomes were volume of SSB, beverage kcal and sugar purchased per transaction.

**Setting:**

Three private schools in Guatemala City, Guatemala.

**Participants:**

Students between 12 and 18 years of age.

**Results:**

Our primary analysis found that the warning poster decreased the overall volume of SSB (in ounces) that adolescents purchased in the warning poster intervention schools (−2·27 oz. 95 % CI = (−2·70, −1·85)) compared with the control school. This reduction was driven by a decrease in SSB purchases (OR = 0·64, 95 % CI = (0·49, 0·86)). The warning posters were associated with a significant reduction in likelihood of purchasing a beverage with kilocalories (calories) (OR = 0·68, 95 % CI = (0·49, 0·92)). These changes were associated with a significant overall decrease in sugar purchased (−5·54 g 95 % CI = (−6·69, −4·39)). The posters were associated with a significant increase in non-SSB purchases in the intervention schools compared with the control school (OR = 1·53, 95 % CI = (1·16, 2·02)).

**Conclusion:**

Our results suggest that messages that warn adolescents about the high-sugar content in SSB may be an effective, low-cost way to modestly reduce purchases of these drinks. These findings provide evidence to support national front-of-package labelling, currently being considered in Guatemala.

Cardiometabolic diseases are rising globally, contributing to a significant proportion of mortality and morbidity worldwide^(1)^. This alarming trend is closely linked to dietary habits, including high consumption of sugar-sweetened beverages (SSB),^(2)^ which are associated with weight gain,^(3)^ increased risk of type 2 diabetes^(4,5)^ and dental caries^(6)^ Adolescents are a priority population for addressing SSB consumption, as they are among the highest consumers of these beverages^(7)^. Furthermore, adolescence is a critical period of physical and psychological development during which new behaviours are established that often persist into adulthood^(8)^. In Latin America, where obesity prevalence is rapidly increasing, SSB consumption remains particularly high^(2)^. For example, a 2010 nationally representative survey in Guatemala found that adults consumed nearly three servings of SSB per day^(3)^. Similarly, among adolescents, a 2015 study of four Guatemala City schools revealed that students drank SSB 2·6 times per week^(9)^.

To address the overconsumption of SSB and other nutritionally poor foods, several countries in the region have implemented front-of-package nutrition warning labels^(10)^. Mandatory labelling initiatives in countries, such as Chile^(11)^, Uruguay^(12)^, Peru^(13)^, Mexico^(14)^ and Argentina,^(15)^ aim to guide healthier choices, especially among younger populations who are highly susceptible to marketing and are key to shaping future trends in public health. Interventions that foster social learning and boost adolescents’ autonomy align with a key developmental need to build a strong sense of competence during a critical period of development^(16)^. Labelling initiatives, therefore, have the potential to alter knowledge and behaviour at an important stage in the formation of eating habits^(17)^.

The Pan-American Health Organisation has called on member states to implement such warning labels as part of a suite of laws and regulations aimed at addressing non-communicable diseases^(18)^. An octagon-shaped nutrition warning label, like the one implemented in Chile, is currently proposed in Guatemala as part of the ‘Law of Healthy Food Promotion’^(19)^. Although Guatemala’s current school nutrition standards provide recommendations for targets by food group, they are less centralised and nutritionally prescriptive compared with school meal programmes in the USA or the UK^(20,21)^.

Front-of-package warning labels have been associated with improved dietary outcomes in both experimental^(22)^ and real-world settings^(23)^, although most of those studies focused on adults, and no real-world studies have been conducted in Guatemala. A meta-analysis of experimental studies, primarily conducted in other Latin American countries, the USA and Canada, found that warning labels lower perceptions of healthfulness and reduce the purchasing and consumption of SSB^(24)^. A narrative review of real-world evaluations of front-of-package food and beverage labels using objective sales data concluded that, on average, such labels lead to small but meaningful reductions in SSB sales^(25)^. A country-wide natural experiment of Chile’s mandated food and beverage warning labels that highlight excessive amounts of calories, Na, added sugars and saturated fats^(11)^ showed a 24 % decrease in household SSB purchases and 10 % decrease in per capita sugar purchases compared with the counterfactual^(23,26,27)^. Chile’s labelling policy, however, was coupled with restrictions on marketing and selling labelled foods to children^(11)^, making it impossible to assess independent warning label effects. A recent crossover randomised experiment among Guatemalan children and adults found that a front-of-package warning label, compared with a Guideline Daily Amount label, improved participants’ understanding of nutrient content and decreased purchase intention and perceptions of healthfulness in products with excessive amounts of specific nutrients^(28)^.

Few labelling studies have focused on adolescents. One online randomised-controlled experiment with 2202 demographically diverse US adolescents found that adolescents chose SSB less often when exposed to warning labels highlighting the health risks of overconsuming SSB (61–65 %) compared with the control group (77 %)^(29)^. Warning labels about sugar and caffeine content decreased purchase intentions in an experimental study among 404 German adolescents, especially for younger compared with older adolescents. However, the warning label was less effective when paired with advertising elements such as a product image, especially among the younger adolescents^(30)^. While hypothetical studies provide valuable insights into adolescent responses to warning labels, real-world experiments are essential for understanding how such interventions function in practical settings. One real-world study randomised corner stores in Baltimore, Maryland, US, to display posters showing information about beverage calories in one of three ways: (1) absolute calorie information, (2) percent daily value or (3) physical activity equivalents for running. Exposure to any nutrition message was associated with a reduced likelihood of adolescents purchasing an SSB compared with a baseline period with no posters^(31)^. A follow-up study randomised corner stores to display posters providing either: (1) absolute calorie information, (2) number of teaspoons of sugar; (3) physical activity equivalents for running or (4) for walking. Exposure to any message was associated with a reduction in SSB purchased by adolescents (59–71 % reduction) compared with a baseline period, with the walking message showing significantly larger reductions than the running message^(32)^.

To contribute to this growing body of evidence, we conducted an experimental study in Guatemala City among three school stores to evaluate the impact of SSB warning messages on adolescent purchasing behaviours. We tested the degree to which posters displaying SSB warning messages and non-SSB promotional messages influenced: (1) the volume (oz) of beverages (SSB and non-SSB) purchased; (2) the number of beverage calories purchased and (3) the amount of sugar from beverages purchased. We hypothesised that Guatemalan adolescents exposed to SSB warning messages would reduce their SSB purchases compared with no intervention.

## Methods

### School selection

To recruit schools for the intervention, we obtained authorisation from the Guatemalan Ministry of Education to approach a convenience sample of twenty-three public schools and four private schools in Guatemala City about the study. Ultimately, we were unable to work with the twenty-three public schools because they were either in areas of high crime, which raised safety concerns for our staff, or they did not have a school store or cafeteria, or the times when the school store or cafeteria were open were so different between them that we could not compare SSB purchases. For these reasons, we conducted the study in the three private secondary schools that agreed to participate. The three schools serve students between the ages of 12 and 18. They are located in three different administrative districts (i.e. *Zona 11*, *Zona 12* and *Zona 15*) within Guatemala City, which are not geographically close to each other. In Guatemala, almost half (48·9 %) of adolescents enrolled in secondary school attend private schools^(33)^, which are predominantly attended by middle- and higher-income urban families^(34)^.

This study was conducted in accordance with the guidelines outlined in the Declaration of Helsinki, and all procedures involving research study participants were approved by the Institutional Review Boards at the University of Pennsylvania (#828762) and the Universidad Francisco Marroquín Department of Medicine (#0017-18). Informed consent was obtained from all participants.

### Intervention assignment

Two of the schools that agreed to participate in the study had a large student population (699 and 700 students), while the other school had 344 students. Although random assignment balances known and unknown confounders only when applied to many units, we used it to determine which of the two larger schools would receive the intervention *v*. the control. We then assigned the smaller school to receive the intervention as well. The large intervention school had one store with two cash registers, where students could purchase items (food, beverages and school supplies) during two recess periods, each with a duration of 30 and 35 min, respectively. The smaller intervention school had one store with one cash register, where students could purchase items for two recess periods of 20 min and 40 min. The large control school had one store with one cash register where students could buy items for three 20-min recess periods. All stores were stocked with a variety of processed, homemade and fresh foods and beverages, which were displayed behind the counter and available for purchase upon request by students. Students did not have other alternative places to purchase food or beverages within or near the schools during the school day. We employed a difference-in-differences approach to compare the combined effect of the two intervention schools with that of the control school. We then examined effects separately for each intervention school.

### Intervention

During the intervention period, schools displayed either a warning poster (in the intervention schools) or a control poster (in the comparison school). Both posters showed six SSB and six no- or low-sugar sweetened beverages (non-SSB) available in the store, along with their calorie counts per serving (see online supplementary material, Supplemental Figure 1). The schools did not permit us to use brand names, so generic names (e.g. soda) were used instead. Beverages with more than 5 g of sugar per 100 ml were considered SSB, consistent with the guideline for the Chilean Law of Food Labelling and Advertisement^(11)^. Non-SSB were those that had 5 g of sugar per 100 ml or less, including those with no sugar content (e.g. plain water and diet drinks). In addition to the nutrition information shown on both posters, the warning poster included octagon-shaped warnings with the words ‘high in sugar’ (in Spanish) next to the six SSB, inspired by the warning labels implemented in several Latin American countries (e.g. Chile, Mexico and Uruguay). The text above the SSB images read: ‘Avoid these beverages because they are high in sugar’. The non-SSB were labelled with a check mark in a circle, and the text read: ‘Drink these beverages instead’. In contrast, the beverages on the control poster had no labels and a single neutral message above all beverages: ‘These beverages are sold in our cafeteria’.

All schools had a four-week baseline assessment period with no posters (8 July–4 August 2019). Then posters were introduced at eye level on the wall next to where students ordered food and beverages at the school stores for the following 4 weeks (5 August–30 August 2019). One poster was displayed at each store (the large intervention school had two stores). We standardised the implementation of the intervention at each store by ensuring it was posted at a similar weight and similar distance to the checkout at each location.

### Data and measures

We collected data from two sources: (1) store transactions and (2) interviewer-administered student survey purchase assessments. We pilot-tested our standardised data collection procedures for 2 weeks at each school store to ensure our trained research assistants could accurately capture all purchases.

#### Store transactions

To collect transaction data, a trained research assistant stood at the cash register at each school store during all recess periods. The research assistant recorded all student purchases in real time on a tablet using the Square Up application (https://squareup.com/), which enables the capture of point-of-sale data. Details about the foods and beverages available for sale (including brand, flavor, size, price and container size) in each school store were pre-programmed into the Square Up application and updated daily. To record a transaction, the research assistant observed each item purchased, selected each item on the tablet and saved the transaction to a virtual storage file.

Our primary outcome was volume of SSB (oz) purchased per transaction based on the transaction data. Secondary outcomes from the transaction data included volume of non-SSB (oz), beverage calories (kcals) and beverage sugar (g) purchased per transaction.

#### Survey purchase data

To collect survey data, research assistants approached all students immediately after they made a purchase at the school store to complete a 5-min survey (see see online supplementary material) throughout the study. Students were eligible to participate in this portion of the study if they gave verbal assent and had not previously completed the survey. Letters were also sent home to parents, informing them of the research and giving their child the opportunity to opt out; however, no parents chose this option. Survey data were collected using the Qualtrics Offline Mobile application for tablets and uploaded to a virtual storage file when a Wi-Fi connection was available.

Survey participants were first asked to list up to six items (foods, beverages and/or school supplies) that they had purchased at the store. Next, participants were asked to rate the healthfulness of four popular drinks, presented in random order (soda, diet soda, water and fruit drink), on a seven-point Likert scale, ranging from 1 (least healthy) to 7 (extremely healthy). Participants were only asked questions about the poster during the intervention, including whether they noticed it (yes/no), and if so, which elements they recalled (i.e. calorie information, information about sugar content of beverages, warning labels, advertising beverages sold at the store and other), whether it influenced their purchase (five-point Likert scale, 1 (least influential) to 5 (most influential)) and how much they trusted the information (five-point Likert scale, 1 (least trustworthy) to 5 (most trustworthy). At both timepoints, one question assessed the usual frequency of SSB consumption (i.e. ‘How many sugary beverages do you usually drink during the week?’ answer options = none, 1 per week, 2–3 per week, 4–5 per week, 1 per day, 2–3 per day, > 3 per day and no answer) and another question assessed the frequency of purchasing at the school store (i.e. ‘With what frequency do you buy products at this store?’ answer options = 1 time per month or less, 2–3 times per month, 1–2 times per week, 3–4 times per week, 1 time per day, more than once per day and no answer). The survey also assessed demographics (gender, age and grade) and self-reported height and weight, which were used to calculate BMI-for-age z-scores using the 2007 WHO Child Growth Standards. Students were given a small prize (i.e. a pen) as appreciation for their participation.

#### Product information

Nutrition and price information were collected for all products sold at the school stores and merged with the transaction and survey data. For packaged foods and beverages, nutrition information was obtained from the Nutrition Facts Panel. Nutrition information from homemade snacks and fruit drinks was estimated using reported serving sizes and the Institute of Nutrition of Central America and Panama Food Composition Table, which includes nutrition information for common prepared foods and beverages (e.g. homemade fruit juice)^(35)^. For those items that did not have a Nutrition Facts Panel or were not found in the Institute of Nutrition of Central America and Panama Food Composition Table, we used the U.S. Department of Agriculture Food Composition Database^(36)^. The nutrition information for each food and beverage item was entered per serving. The total nutritional content of each item per container was then calculated using the number of servings per container.

### Statistical analyses

In both the transaction data and the survey purchase assessment data, we excluded all beverage outliers that were more than three standard deviations from the mean beverage volume (oz) purchased (e.g. purchases of large beverages not intended for individual consumption but for another purpose, such as a class party, as noted in field notes).

**Figure 1.**
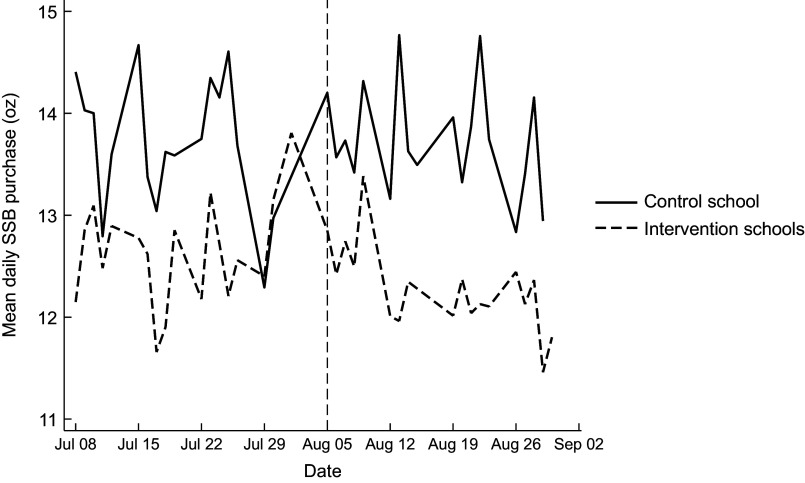
Mean daily SSB purchases trends (oz) by school arm. SSB, sugar-sweetened beverage.

We plotted the ounces of SSB purchased (primary outcome) in the combined intervention schools *v*. the control school. Visual inspection of the data demonstrated that they meet the parallel trends assumption for a difference-in-differences analysis (see Figure 1).

The primary analysis used a two-part model, with a difference-in-differences specification to estimate the change from baseline to the end of the warning poster period in the intervention schools compared with the control school. In addition, we conducted logistic regression models to assess the odds of purchasing beverages across all transactions. We used a two-part model for our primary analysis, rather than a generalised linear model, because it accounted for the fact that many students did not purchase an SSB or a beverage with kcals and therefore had zeros for that outcome. The two-part model estimated a joint significance for the entire sample as well as the OR for the likelihood that a beverage (i.e. SSB or non-SSB) was purchased. It then used a linear model to estimate the volume of SSB or non-SSB purchased, conditional on buying one of those beverages. We also ran two-part models for the beverage calories and sugar outcomes. The model specification for our primary analyses was

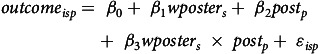

where 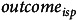 was a measure per purchase *i* in school *s* during period *p*. 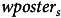 was an indicator variable for whether school *s* was allocated to the control (0) or the warning (1) poster condition. 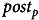 takes the value of one in the intervention period (after August 5th) and zero in the baseline period. 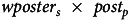 is the interaction between those predictors. Therefore, 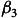 is the coefficient of interest, which captures the average effect of the warning poster compared with the control poster during the intervention period relative to baseline. Fixed effects at the school and individual levels are part of the error term 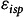 . For the survey purchase assessments, we used the same analytic approach but also included students’ age and gender as covariates, which were not available for the transaction data. We treated our secondary perception outcomes as continuous and used a GLM. All statistical analyses were conducted using Stata 18.

## Results

### School store transactions

We recorded 16 659 transactions at the three schools, comprising 25 586 food, beverage and other items. After excluding beverage outlier transactions (*n* 57, 0·34 %), we analysed 16 602 purchase transactions (24 743 items). Analyses were conducted at the beverage transaction level.

#### Baseline beverage transactions

The median number of items per transaction was 1 (Mean = 1·49, sd = 0·79, range 1–17). Most transactions included foods (*n* 14 366, 86·5 %), while 33·9 % (*n* 5624) included beverages. Only a few included other items (e.g. school supplies) (*n* 19, 0·1 %). Most beverage transactions included SSB (*n* 2192, 81·8 % of 2679 baseline beverage purchases). The average expenditure per purchase was 5·84 ± 2·21 Quetzales (Q to US$ exchange rate on February 2025, Q1 = US$0·13). The average SSB ounces per beverage transaction was 10·7 ± 6·2 oz. Mean beverage calories were 124 ± 81 per beverage transaction, while mean grams of beverage sugar were 28 ± 18. The proportion of purchases with a beverage across the study periods remained the same in the control (from 33·4 % to 32·3 %, OR = 0·95, 95 % CI = (0·86, 1·05)) and significantly increased in the warning poster (from 32·9 % to 36·3 %, OR = 1·15, 95 % CI = (1·06, 1·26)) randomised schools (Table 1). Across all schools, the most commonly purchased beverages were carbonated drinks (46·8 % of beverage purchases), fruit drinks (17·3 %), bottled water (10·9 %) and homemade drinks (8·2 %). Drink offerings and average spending per transaction were similar across schools and periods.

**Table 1.** Descriptive statistics for all transactions by period and school arm

	Control				Warning poster intervention			
	Pre		Post		Pre		Post	
Total transactions	3556		3818		4516		4712	
Item counts	4792		4959		7360		7632	
Number of transactions with^†^	*n*	%	*n*	%	*n*	%	*n*	%
Any beverage	1189	33·4	1235	32·3	1490	32·9	1710	36·3
SSB % beverage transactions	991	83·3	1066	86·3	1200	80·5	1320	77·2
Non-SSB % Beverage transactions	198	16·7	169	13·7	306	20·5	408	23·9
Food	3020	84·9	3243	84·9	3991	88·4	4112	87·3
Beverages smaller than 12 oz	238	20·2	170	13·8	511	34·3	516	30·2
Means per beverage transaction	Mean	sd	Mean	sd	Mean	sd	Mean	sd
Beverage items	1·0	0·2	1·0	0·1	1·0	0·2	1·0	0·2
Beverage oz	15·1	5·6	14·8	5·1	13·5	4·5	13·4	4·6
SSB oz	11·4	6·4	11·9	5·9	10·2	5·9	9·5	5·9^*^
Non-SSB oz	3·7	8·7	2·9	7·7^*^	3·5	7·5	3·9	7·7
Beverage kcals	133	88	127	75	117	74	105	72^*^
Beverage sugar (g)	30	18	30	16	27	17	24	16^*^
Beverage spending in Quetzales	6·09	2·26	5·91	2·10^*^	5·65	2·15	5·49	2·06^*^

SSB, sugar-sweetened beverage.

* *P* < 0·05.

† Transactions could include more than one item, so each row of transaction counts is not mutually exclusive.

**Figure 2.**
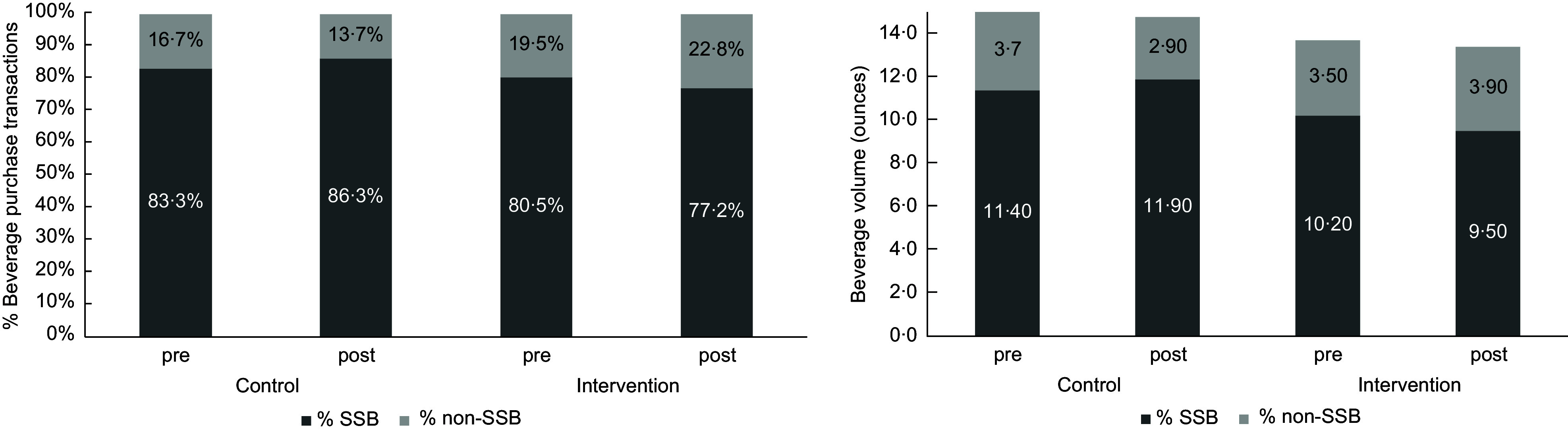
Unadjusted frequency and mean volume (oz) of purchase beverage transactions by period and school.

Figure 1 presents the mean daily SSB purchase trends by intervention and control schools. As summarised in Table 2, the two-part model revealed that the warning posters led to a significant overall decrease of 2·27 oz of SSB purchased from baseline in the warning poster (intervention) schools compared with the control school. This reduction was driven by a decline in SSB purchases (OR = 0·64, 95 % CI = (0·49, 0·86)). Among those purchasing an SSB, there was no significant change in the volume purchased. Consistent with this effect, there was a significant reduction in the likelihood of purchasing a beverage with calories (OR = 0·68, 95 % CI = (0·49, 0·92)), but among those purchasing beverages with calories, there was no significant change in calories (1·28 (−5·69, 8·25)). This translated to a significant overall reduction of 7·17 calories from beverages (95 % CI = (−10·52, −3·83)) in the intervention schools compared with the control school. Similarly, the warning posters were associated with a significant overall decrease of 5·54 g of sugar from beverages (95 % CI = (−6·69, −4·39)). This decrease was driven by a significant decrease in the likelihood of purchasing a beverage with sugar (OR = 0·64, 95 % CI = (0·49, 0·86)), and no significant change in the amount of sugar purchased among those purchasing a beverage with sugar. Figure 2 presents the unadjusted percentage of beverage purchase transactions with SSB and mean SSB volume (oz) by period (baseline and intervention) and school.

**Table 2:** Beverage transactions, two-part models with difference-in-differences interaction term

Outcomes	Control (*n*_bev_ = 2424)	Warning poster intervention (*n*_bev_ = 3200)	
		b/OR	95 % CI
Beverage volume^†^			
Beverage oz purchased, b (95 % CI)	Ref	0·16	–0·36, 0·68
SSB volume			
Purchased SSB, OR (95 % CI)	Ref	0·64	0·49, 0·86^*^
SSB oz if purchased SSB, b (95 % CI)	Ref	−0·32	–0·75, 0·12
Two-part model overall effect	Ref	−2·27	–2·70, –1·85
Two-part model estimate	Ref	Chi^2^(2) = 10·82^*^	
Non-SSB volume			
Purchased non-SSB, OR (95 % CI)	Ref	1·53	1·16, 2·02^*^
SSB oz if purchased non-SSB, b (95 % CI)	Ref	1·25	0·42, 2·07^*^
Two-part model overall effect	Ref	0·79	0·24, 1·35
Two-part model estimate	Ref	Chi^2^(2) = 9·35^*^	
Beverage calories			
Purchased beverage with calories, OR (95 % CI)	Ref	0·68	0·49, 0·92^*^
Calories from beverages if purchased calories, b (95 % CI)	Ref	1·28	–5·69, 8·25
Two-part model overall effect	Ref	−7·17	–10·52, –3·83
Two-part model estimate	Ref	Chi^2^(2) = 6·36^*^	
Beverage sugar			
Purchased beverage with sugar, OR (95 % CI)	Ref	0·64	0·49, 0·86^*^
Sugar from beverages if purchased sugar, b (95 % CI)	Ref	−0·96	–2·32, 0·39
Two-part model overall effect	Ref	−5·54	–6·69, –4·39
Two-part model estimate	Ref	*χ* ^2^(2) = 10·80^*^	

SSB, sugar-sweetened beverages.

All analyses are at the transaction level. If more than one beverage was purchased in a transaction, the sum was used for these analyses. *n* 40 transactions included purchases of both SSB and non-SSB. Baseline period and the control school were the reference categories in all analyses. The *n*s for the regression part of the two-part models were smaller than the other models because they were conditional on > 0 SSB ml purchased, > 0 non-SSB ml purchased, > 0 beverage kcals purchased and > 0 beverage sugar purchased, respectively. See Table 1 for marginal means.

* *P* < 0·05.

† The model for beverage volume is not a two-part model because this analysis is limited to beverage transactions.

The warning posters were also associated with a significant increase in the likelihood of purchasing a non-SSB in the intervention schools compared with the control school (OR = 1·53, 95 % CI = (1·16, 2·02)). This translated to a significant overall increase in the volume purchased of 0·79 oz. 95 % CI = (0·24, 1·35) of non-SSB.

### Students’ purchase assessments and surveys

We approached 1168 students across the three schools during the study period to complete purchase assessments and surveys. Out of 832 unique students approached to do the study, 794 consented to participate (see online supplementary material, Supplemental Figure 2). After excluding outlier transactions (*n* 2, 0·25 %), we analysed 792 purchase assessments.

The students’ ages, the number of SSB they consumed per day and the number of times per day that they made purchases in the store were similar across schools. Statistical models adjusted for age and self-reported gender because the proportion of female students and age were imbalanced across schools (Table 3).

**Table 3.** Sample of student purchase assessments, characteristics by period and school arm^*^

	Control						Warning poster intervention					
	Pre			Post			Pre			Post		
	% miss	Mean	sd	% miss	Mean	sd	% miss	Mean	sd	% miss	Mean	sd
Number of observations		165			143			225			259	
Age	0	15·4	1·6	0	15·0	1·5	0	15·0	1·6	0	15·4	1·5
		*n*	%		*n*	%		*n*	%		*n*	%
Female	0	57	34·6	0	46	32·2	0	106	47·1	0	142	54·8
Grade												
1ero básico	25·5	32	26·0	0	33	23·8	16·9	47	20·9	0·4	58	22·4
2ndo básico		22	17·9		46	32·2		46	20·4		45	17·4
3ero básico		19	15·5		23	16·1		32	14·2		48	18·5
4to bachillerato		20	16·3		22	15·4		35	15·6		71	27·4
5to bachillerato		30	24·4		18	12·6		27	12·0		36	13·9
		Mean	sd		Mean	sd		Mean	sd		Mean	sd
BMI-for-age Z-score^†^	49·1	0·32	0·95	49·7	0·23	1·02	42·7	0·08	0·91	52·5	0·68	0·79
SSB consumed per day	0·6	0·7	0·7	0	0·6	0·6	0	0·8	0·9	0	0·7	0·9
Store purchases (times/day)	0	0·5	0·5	0·7	0·4	0·4	0	0·4	0·4	0·4	0·3	0·4

SSB, sugar-sweetened beverage.

% miss = % missing data.

* Unadjusted raw means.

† BMI-for-age Z-score according to 2007 WHO Child Growth Standards.

The student purchases included approximately the same number of items per transaction (Mean = 1·72, sd = 0·92, range 1–6) as the overall transactions in each school (Mean = 1·49, sd = 0·79, range 1–17). However, a greater proportion of student purchase assessments came from students who purchased SSB (36·7 %) relative to the proportion of all transactions that included SSB (27·6 %), indicating this student sample is biased towards SSB consumers. Our transaction data analysis provides a more rigorous assessment of the warning posters’ influence on beverage sales, as it captures all sales at the school stores. Therefore, we only include secondary outcomes from the survey in the main paper. Results from beverage purchases, as assessed by the surveys, are presented in online supplementary material, Supplemental Tables 1 and 2 for completeness. Supplemental Figure 3 includes the frequency and mean volume of purchase assessments.

The students in the schools assigned to the warning poster reported a significantly greater influence of the poster on their purchases (mean rating of 2·5 on a 5-point scale, Table 4) compared with the students viewing the control poster (mean rating of 2·0). The warning poster did not influence the perception of healthfulness of water, fruit drink, Coke Zero or Coke, in the intervention *v*. control schools. Unexpectedly, the warning poster decreased the odds of recalling calories in beverages (OR = 0·57, 95 % CI = (0·38, 0·85)) compared with the control poster. However, the warning posters significantly increased the odds of identifying intervention-specific information (OR = 18·78, 95 % CI = (7·93, 44·44)), like the sugar content of beverages (24 % *v*. 11 %) and the warning labels (20 % *v*. 0 %).

**Table 4.** Sample of purchase assessments adjusted for gender and age, descriptive statistics by period and school arm

	Control (*n* 308)		Intervention (*n* 484)			
	Pre	Post	Pre	Post	Estimate	
	Mean or %	Mean or %	Mean or %	Mean or %	DID	95 % CI
Beverage healthfulness^*^						
Water	6·7	6·8	6·9	6·8	−0·03	–0·24, 0·19
Fruit drink	4·2	4·2	4·2	3·8	−0·34	–0·77, 0·09
Coke Zero	2·8	3·2	2·8	2·9	0·21	–0·66, 0·23
Coke	1·9	1·9	1·8	1·9	0·32	–0·04, 0·68
Post-intervention only…		(*n* 143)		(*n* 259)	OR	95 % CI
Noticed the poster	–	60·8 %	–	60·2 %	0·95	0·62, 1·47
					b	95 % CI
Trust in the poster information^†^	–	3·5	–	3·7	0·15	–0·08, 0·39
If noticed…		(*n* 86)		(*n* 155)	OR	95 % CI
Recalled contents of poster	–	85·8 %	–	90·3 %	1·34	0·62, 2·90
					b	95 % CI
Poster influenced purchase^‡^	–	2·0	–	2·5	**0·46**	**0·12, 0·81**
If recalled…describe the poster		(*n* 225)		(*n* 259)	OR	95 % CI
Calorie information	–	85·1 %	–	26·6 %	**0·57**	**0·38, 0·85**
Advertising bevs sold	–	22·2 %	–	5·8 %	0·49	0·23, 1·00
Intervention-specific information	–	10·8 %	–	69·2 %	**18·78**	**7·93, 44·44**
Sugar content of bevs	–	10·8 %	–	24·3 %	**5·14**	**2·40, 11·02**
Stop sign	–	0 %	–	20·1 %	NA	
Other	–	2·6 %	–	5·0 %	4·19	0·99, 17·88

DID, difference in differences.

All analyses are at the purchase assessment level and adjust for gender and age. Baseline and the control school were the reference categories in all DID analyses. Control estimates are from primary contrast with the intervention school. For bold values, *P* < 0·05.

* Likert scale from 1 to 7, 1 being the least and 7 the most healthy.

† Likert scale from 1 to 5, 1 being the least and 5 the most trustful.

‡ Likert scale from 1 to 5, 1 being the least and 5 the most influential.

## Discussion

In this study, we found that displaying a poster warning about the high-sugar content of SSB at school stores was associated with a decrease in the number of SSB ounces adolescents purchased when compared with a control school. This translated into a small reduction in the purchase of beverage calories, but a meaningful reduction of five grams of sugar purchased per transaction. This represents 20 % of the daily added sugar limit recommended for adolescents^(37)^. These differences in purchasing behaviour emerged despite no changes in reported perceptions of the healthfulness of SSB in a sub-sample of students. The warning posters were, however, associated with an increase in accurately recalling the sugar content of SSB.

Our results that the warning poster intervention decreased the overall volume of SSB purchased are consistent with previous studies that found that presenting information about the nutritional content of foods or beverages in the form of posters in stores can persuade consumers to make healthier choices^(30–32)^. In addition, a recent before-and-after evaluation of Chile’s Law of Food Labelling and Advertising, which requires warning labels on foods when they are high in calories, total sugar, Na and saturated fat, found that SSB purchased volume decreased by 23·7 % and non-SSB purchased volume increased by 4·8 %^(26)^. Another study evaluating household beverage purchasing trends in Peru, after the implementation of an SSB warning label policy at the national level, found a decline in SSB volume purchase of 21 ml per capita per month^(13)^. Furthermore, studies that have examined beverage purchases in experimental settings have also found that warning labels have the potential to reduce SSB purchases. A recent study that took place in a convenience store laboratory found that pictorial health warning labels in SSB lowered the likelihood of buying a sugary drink by 17 percentage points, compared with control labels (barcode image)^(22)^. Another study in the same convenience store laboratory found that these labels decreased the purchased SSB calories by 22 %^(38)^. Our study adds to this body of evidence that warning messages have the potential to help consumers make healthier choices.

The warning posters in the intervention schools did not influence health perceptions. This evidence is not consistent with other studies showing that warning labels work, in part, by correcting misperceptions that certain drinks, such as fruit or sports drinks, are healthy^(39)^. In Chile, there is evidence that warning labels have been especially effective for products that consumers consider to be relatively healthy, like breakfast cereals^(27)^ and an experiment found that warning labels reduced perceptions of healthfulness of an orange fruit drink^(40)^.

### Strengths and limitations

This study has several limitations. First, this study only included three schools, which limits its generalisability. Second, we tested warning posters but were not permitted by the schools to test labels displayed directly on products, which may produce larger effects over time. Additional studies are needed to test the effects of warning labels on products in school stores. Third, the presence of research assistants collecting data at the point-of-sale may have made students more conscious of their beverage purchases. Although the same data collection procedures were used at the control and intervention schools, the warning messages may have heightened the researchers’ salience. Fourth, our measures of height and weight were self-reported, which introduces bias, but we only used it descriptively, not as an outcome.

Strengths of this study include the use of a natural experiment that analysed objective sales data. Another strength is that this is one of the only studies to examine a nutrition intervention in school settings in Guatemala. Furthermore, most studies assessing point-of-purchase interventions to change food or beverage choices have been conducted in supermarkets or grocery stores, rather than schools. Only a few have focused on interventions designed to discourage selection of unhealthy products, rather than encouraging healthy food choices. We also tested a warning label on the posters that is recommended by the PAHO and has been implemented in several countries in the region. It would, however, be valuable to test different warning label designs, particularly given that many different languages are spoken within Guatemala, underscoring the need for symbols that are easily understood regardless of language and literacy level.

Our results suggest that messages warning adolescents about the high-sugar content in SSB may be an effective way to reduce their purchases of SSB. These findings provide initial evidence in support of the ‘Law of Healthy Food Promotion’^(19)^, currently being considered in the Guatemalan Congress and offer schools a cost-effective and easy-to-implement intervention.

## Supporting information

Chacón et al. supplementary material Chacón et al. supplementary material

## Supplementary material

For supplementary material accompanying this paper visit https://doi.org/10.1017/S1368980025101638
