# Supplementary material for: Point-of-sale sugar-sweetened beverage warning posters v. control posters were associated with reductions in school store sugar-sweetened beverage purchases made by Guatemalan adolescents
Source: Public Health Nutr. 2026 Jan 2;29(1):e24. doi: 10.1017/S1368980025101638 (PMC12886556; doi:10.1017/S1368980025101638)
Supplement: Chacón et al. supplementary material [file S1368980025101638sup001.docx]

**Supplementary Material:**

**Supplementary Figure 1. Treatment and Control Posters**

**Supplementary material: Intercept survey**

| Date: | Time: | | | | | Day of week: | | | | | Interviewer name: | | | | | | | | | School Name: | | | | | | | |
| --- | --- | --- | --- | --- | --- | --- | --- | --- | --- | --- | --- | --- | --- | --- | --- | --- | --- | --- | --- | --- | --- | --- | --- | --- | --- | --- | --- |
| **CONSENT SCRIPT:** We are asking you to take this survey because you just made a purchase at your school store. If you agree to take the survey, we will ask you some questions about what you purchased. It should take about 5 minutes. We will not document any personal details that will connect your answers with your name. All of your answers will be kept private and no one outside of our research team will be able to see them.There are no risks to participating in this study. We will give you a small prize at the end of the survey for your participation. You do not have to join this study. If you say okay now and you change your mind, you can stop answering the questions at any time. Do you have any questions? Do you agree to take the survey? (circle one) : Yes No | | | | | | | | | | | | | | | | | | | | | | | | | | | |
| How often do you shop at this store? | | | | | 1 time per month or less | | | 2-3 times per month | | | | | 1-2 times per week | | | | How many times did you shop at this store today? | | | | | | 1 | | | 2 | |
|  | | | | | 3-4 times per week | | | 1 time per day | | | | | More than 1 time per day | | | | How much did you spend on the first visit? | | | | | |  | | |  | |
| Was this purchase only for you, to share, or for someone else? | | | | | Only you  Share | | | Someone else  Refused | | | | |  | | | | If student shopped twice, How much did you spend on the second visit? | | | | | |  | | |  | |
| Was this second purchase only for you, to share, or for someone else? | | | | | | | | | | | Only you | | | Share | | | Someone else Refused | | | | | | | | | | |
| What type of item was Purchase A? | | Drink | | Food | | | If food, What type of food was purchased? | | | | | | | | | Brand: | | Type: | | | Flavor: | | | |  | | |
| Please select Purchase A: | |  | |  | | |  | | | | | | | | |  | |  | | |  | | | |  | | |
| Pepsi | | 237 | |  | | | Petit Fruta Fresca Frutas Tropicales | | | | | | | | | 500 | |  | | | H2O2 limoneto | | | | 600 | | |
| Pepsi | | 355 | |  | | | Petit Fruta Fresca Manzana y Uva | | | | | | | | | 500 | |  | | | Aqua Eco Agua | | | | 750 | | |
| Pepsi | | 600 | |  | | | Gatorade Naranja | | | | | | | | | 600 | |  | | | hot cocoa mix nestle | | | | 237 | | |
| Mirinda Naranja | | 237 | |  | | | Gatorade Uva | | | | | | | | | 600 | |  | | | Flavor 1 | | | | 473 | | |
| 7 up | | 237 | |  | | | Be Light Limon | | | | | | | | | 237 | |  | | | Flavor 2 | | | | 473 | | |
| 7 up | | 355 | |  | | | Be Light Jamaica | | | | | | | | | 237 | |  | | | Flavor 3 | | | | 473 | | |
| Mountain Dew | | 355 | |  | | | H2O2 naranchelo | | | | | | | | | 600 | |  | | | Etc. | | | | 473 | | |
| Petit Nectar de Durazno | | 150 | |  | | |  | | | | | | | | |  | |  | | |  | | | |  | | |
|  | |  | |  | | |  | | | | | | | | |  | |  | | | Quantity of Purchase A: | | | |  | | |
| Did the student purchase a **second** item? Yes No | | | | | | | | | | | | | | | |  |  |  |  |  |  |  |  |  |  |  |  |
| What type of item was Purchase B? | | Drink | | Food | | | If food, what type of food was purchased? | | | | | | | | | Brand: | | Type: | | | Flavor: | | | |  | | |
| Please select Purchase B: | |  | |  | | |  | | | | | | | | |  | |  | | |  | | | |  | | |
| Pepsi | | 237 | |  | | | Petit Fruta Fresca Frutas Tropicales | | | | | | | | | 500 | |  | | | H2O2 limoneto | | | | 600 | | |
| Pepsi | | 355 | |  | | | Petit Fruta Fresca Manzana y Uva | | | | | | | | | 500 | |  | | | Aqua Eco Agua | | | | 750 | | |
| Pepsi | | 600 | |  | | | Gatorade Naranja | | | | | | | | | 600 | |  | | | hot cocoa mix nestle | | | | 237 | | |
| Mirinda Naranja | | 237 | |  | | | Gatorade Uva | | | | | | | | | 600 | |  | | | Flavor 1 | | | | 473 | | |
| 7 up | | 237 | |  | | | Be Light Limon | | | | | | | | | 237 | |  | | | Flavor 2 | | | | 473 | | |
| 7 up | | 355 | |  | | | Be Light Jamaica | | | | | | | | | 237 | |  | | | Flavor 3 | | | | 473 | | |
| Mountain Dew | | 355 | |  | | | H2O2 naranchelo | | | | | | | | | 600 | |  | | | Etc. | | | | 473 | | |
| Petit Nectar de Durazno | | 150 | |  | | |  | | | | | | | | |  | |  | | | Quantity of Purchase B: | | | |  | | |
| Did the student purchase a **third** item? Yes No | | | | | | | | | |  |  |  |  |  |  |  |  |  |  |  |  |  |  |  |  |  |  |
| What type of item was Purchase C? | | Drink | | Food | | | If food, what type of food was purchased? | | | | | | | | | Brand: | | Type: | | | Flavor: | | | |  | | |
| Please select Purchase C: | |  | |  | | |  | | | | | | | | |  | |  | | |  | | | |  | | |
| Pepsi | | 237 | |  | | | Petit Fruta Fresca Frutas Tropicales | | | | | | | | | 500 | |  | | | H2O2 limoneto | | | | 600 | | |
| Pepsi | | 355 | |  | | | Petit Fruta Fresca Manzana y Uva | | | | | | | | | 500 | |  | | | Aqua Eco Agua | | | | 750 | | |
| Pepsi | | 600 | |  | | | Gatorade Naranja | | | | | | | | | 600 | |  | | | hot cocoa mix nestle | | | | 237 | | |
| Mirinda Naranja | | 237 | |  | | | Gatorade Uva | | | | | | | | | 600 | |  | | | Flavor 1 | | | | 473 | | |
| 7 up | | 237 | |  | | | Be Light Limon | | | | | | | | | 237 | |  | | | Flavor 2 | | | | 473 | | |
| 7 up | | 355 | |  | | | Be Light Jamaica | | | | | | | | | 237 | |  | | | Flavor 3 | | | | 473 | | |
| Mountain Dew | | 355 | |  | | | H2O2 naranchelo | | | | | | | | | 600 | |  | | | Etc. | | | | 473 | | |
| Petit Nectar de Durazno | | 150 | |  | | |  | | | | | | | | |  | |  | | | Quantity of Purchase C: | | | |  | | |
| **I am now going to ask you about the healthiness of the item(s) you purchased.** | | | | | | | | | | | | | | | | | | | | | | | | | | |  |
| On a scale of 1-5, with 1 being the least healthy and 5 being extremely healthy, how healthy would you rate Purchase A? | | | | | | | | | | | | | | | | | | | | | | | | | | | |
| Not at all healthy (1) | | | 2 | | | | | | | | | Neither healthy nor unhealthy (3) | | | | | | | 4 | | | | | Extremely healthy (5) | | | |
| On a scale of 1-5, with 1 being the least healthy and 5 being extremely healthy, how healthy would you rate Purchase A? | | | | | | | | | | | | | | | | | | | | | | | | | | |  |
| Not at all healthy (1) | | | 2 | | | | | | | | | Neither healthy nor unhealthy (3) | | | | | | | 4 | | | | | Extremely healthy (5) | | | |
| On a scale of 1-5, with 1 being the least healthy and 5 being extremely healthy, how healthy would you rate Purchase A? | | | | | | | | | | | | | | | | | | | | | | | | | | |  |
| Not at all healthy (1) | | | 2 | | | | | | | | | Neither healthy nor unhealthy (3) | | | | | | | 4 | | | | | Extremely healthy (5) | | | |
|  | | |  | | | | | | | | | | |  | | | | |  | | | | |  | | | |
| Did you notice any nutrition-related posters while you were shopping today? | | | | | | | | | | | | | | | Yes | | | | No | | | | |  | | | |
| If yes, can you recall what any of the nutrition-related posters said? | | | | | | | | | | | | | | | Yes | | | | No | | | | |  | | | |
| Please describe what the posters said (do not show options to the participant. Participant answers do not have to align precisely with choice, as long as the general idea is the same) | | | | | | | | | | | | | | | Calorie information | | | | | Information about sugar content of beverages | | | | | Telling you what to drink and what not to drink (black stop signs) | | |
|  |  |  |  |  |  |  |  |  |  |  |  |  |  |  | Advertising beverages sold at the store | | | | | Other | | | | |  | | |
| If you saw this poster, do you think it influenced what you purchased? | | | | | | | | | | | | | | | Yes | | | | No | | | | |  | | | |
| How many sugary drinks do you usually drink during one week? This includes soda, sweetened iced tea, sports drinks, or juice. | | | | | | | | | | | | | | | None | | | | 1 per week | | | | | 2-3 per week | | | |
|  | | | | | | | | | | | | | | | 4-5 per week | | | | 1 per day | | | | | More than 1 per day | | | |
|  | | | | | | | | | | | | | | | Don’t know | | | | Refused | | | | |  | | | |
| **If store includes a poster, show the image of the poster now.** | | | | | | | | | | | | | | |  | | | |  | | | | |  | | | |
| How much do you trust the information on this poster? | | | | | | | | | Not at all (1) | | | | | 2 | Neither trust nor distrust (3) | | | | | | | 4 | | | A lot (5) | | |
| **DEMOGRAPHIC QUESTIONS** | | | | | | | | | | | | | | | Gender: | | | | Age: | | | | |  | | | |
| Grade level: | | | | | | | | | | | | | | | Height (meters): | | | | Weight (pounds): | | | | |  | | | |
| Comments: | | | | | | | | | | | | | | |  | | | |  | | | | | **END OF SURVEY** | | | |

**Supplementary Figure 2.** Study Participants

Summaries of the purchase assessment transaction data are included in **Supplementary Table 1**. In this purchase assessment data, there was no significant effect on SSB ounces, non-SSB ounces, beverage calories, or beverage sugars purchased from baseline among beverage transactions in the intervention schools compared to control (**Supplementary Table 2a**).

| **Supplementary Table 1. Student purchase assessments, descriptive statistics by period and school arm** | | | | |
| --- | --- | --- | --- | --- |
|  | **Condition** | | | |
|  | **Control school (n=308)** | | **Intervention schools (n= 484)** | |
|  | **Pre** | **Post** | **Pre** | **Post** |
| Total purchases | 165 | 143 | 225 | 259 |
| Item counts | 243 | 206 | 438 | 484 |
| Number of purchases with ^a^ | n (%) | n (%) | n (%) | n (%) |
| Any beverage | 74 (44.9) | 48 (33.6) | 96 (42.7) | 131 (50.6) |
| SSBs % Beverage purchases | 63 (85.1) | 43 (89.6) | 79 (82.3) | 106 (80.9) |
| Non-SSBs % Beverage purchases | 11 (14.9) | 5 (10.4) | 17 (17.7) | 25 (19.1) |
| Food | 135 (81.8) | 121 (84.6) | 208 (92.4) | 227 (87.6) |
| Means per purchase ^b^ | M+SD | M+SD | M+SD | M+SD |
| Among beverage purchases |  |  |  |  |
| Beverage items | 1.1 ± 0.3 | 1.1 ± 0.3 | 1.0 ± 0.2 | 1.1 ± 0.2 |
| Beverage oz, if beverage purchase | 16.4 ± 6.8 | 14.8 ± 5.9 | 14.2 ± 5.3 | 14.1 ± 5.0 |
| SSB oz | 13.1 ± 8.1 | 12.6 ± 6.9 | 10.8 ± 6.5 | 10.8 ± 6.4 |
| Non-SSB oz | 3.3 ± 8.3 | 2.2 ± 6.6 | 3.3 ± 7.3 | 3.3 ± 7.0 |
| Beverage kcals, if beverage purchase | 139 ± 86 | 136 ± 76 | 119 ± 74 | 119 ± 73 |
| Beverage sugar (g), if beverage purchase | 33 ± 20 | 33 ± 18 | 28 ± 17 | 27 ± 16 |
| Among all purchases |  |  |  |  |
| Total spending in Quetzales | 10.59 ± 5.47 | 9.45 ± 5.87 | 11.52 ± 7.42 | 10.75 ± 6.78 |
| ^a^ Purchases could include more than one item, so each row of purchase counts is not mutually exclusive. | | | | |

| **Supplementary Table 2. Student purchase assessments, two-part models with difference-in-differences interaction term (adjusted for gender and age)** | | | |
| --- | --- | --- | --- |
| **Outcomes** | **Control (n=122)** | **Intervention (n=304)** |  |
| **Beverage volume^a^** |  |  |  |
| Beverage oz purchased, b [95% CI] | Ref | 1.50 [-1.02, 4.03] |  |
| **SSB volume** |  |  |  |
| Purchased SSB, OR [95% CI] | Ref | 1.13 [0.26, 4.94] |  |
| SSB oz if purchased SSB, b [95% CI] | Ref | 0.20 [-2.35, 2.76] |  |
| Two-part model overall effect | Ref | -1.31 ± 1.12 |  |
| Two-part model estimate | Ref | Chi^2^(2) = 0.07 |  |
| **Non-SSB volume** |  |  |  |
| Purchased non-SSB, OR [95% CI] | Ref | 1.17 [0.28, 4.92] |  |
| SSB oz if purchased non-SSB, b [95% CI] | Ref | 0.16 [-3.31, 3.64] |  |
| Two-part model overall effect | Ref | 0.56 ± 1.24 |  |
| Two-part model estimate | Ref | Chi^2^(2) = 0.06 |  |
| **Beverage calories** |  |  |  |
| Purchased beverage, OR [95% CI] | Ref | 1.07 [0.23, 4.94] |  |
| Calories from beverages if purchased SSB, b [95% CI] | Ref | 3.78 [-26.89, 34.46] |  |
| Two-part model overall effect | Ref | -11.61 ± 12.62 |  |
| Two-part model estimate | Ref | Chi^2^(2) = 0.07 |  |
| **Beverage sugar** |  |  |  |
| Purchased SSB, OR [95% CI] | Ref | 1.13 [0.26, 4.94] |  |
| Sugar from SSBs if purchased SSB, b [95% CI] | Ref | -1.23 [-8.04, 5.57] |  |
| Two-part model overall effect | Ref | -5.05 ± 2.96 |  |
| Two-part model estimate |  | Chi^2^(2) = 0.17 |  |
| All analyses are at the purchase assessment-level and adjusted for gender and age. If more than one beverage was purchased by a participant, the sum was used for these analyses. Baseline and the control school were the reference categories in all analyses. The *n*s for the regression part of the two-part models were much smaller than the other models because they were conditional on >0 mL purchased, >0 kcals purchased, and >0 sugar purchased, respectively.  ^a^ The model for Beverage volume is not a two-part model because this analysis is limited to beverage transactions.  p<0.05 | | | |
